# Molecular codes defining rostrocaudal domains in the embryonic mouse hypothalamus

**DOI:** 10.3389/fnana.2015.00046

**Published:** 2015-04-17

**Authors:** José L. Ferran, Luis Puelles, John L. R. Rubenstein

**Affiliations:** ^1^Department of Human Anatomy and Psychobiology, School Medicine, University of Murcia and IMIB (Instituto Murciano de Investigación Biosanitaria)Murcia, Spain; ^2^Department of Psychiatry, Rock Hall, University of CaliforniaSan Francisco, CA, USA

**Keywords:** peduncular hypothalamus, terminal hypothalamus, acroterminal domain, genoarchitecture

## Abstract

The prosomeric model proposes that the hypothalamus is a rostral forebrain entity, placed ventral to the telencephalon and rostral to the diencephalon. Gene expression markers differentially label molecularly distinct dorsoventral progenitor domains, which represent continuous longitudinal bands across the hypothalamic alar and basal regions. There is also circumstantial support for a rostrocaudal subdivision of the hypothalamus into transverse peduncular (caudal) and terminal (rostral) territories (PHy, THy). In addition, there is evidence for a specialized acroterminal domain at the rostral midline of the terminal hypothalamus (ATD). The PHy and THy transverse structural units are presently held to form part of two hypothalamo-telencephalic prosomeres (hp1 and hp2, respectively), which end dorsally at the telencephalic septocommissural roof. PHy and THy have distinct adult nuclei, at all dorsoventral levels. Here we report the results of data mining from the Allen Developing Mouse Brain Atlas database, looking for genes expressed differentially in the PHy, Thy, and ATD regions of the hypothalamus at several developmental stages. This search allowed us to identify additional molecular evidence supporting the postulated fundamental rostrocaudal bipartition of the mouse hypothalamus into the PHy and THy, and also corroborated molecularly the singularity of the ATD. A number of markers were expressed in Thy (*Fgf15, Gsc, Nkx6.2, Otx1, Zic1/5*), but were absent in PHy, while other genes showed the converse pattern (*Erbb4, Irx1/3/5, Lmo4, Mfap4, Plagl1, Pmch*). We also identified markers that selectively label the ATD (*Fgf8/10/18, Otx2, Pomc, Rax, Six6*). On the whole, these data help to explain why, irrespective of the observed continuity of all dorsoventral molecular hypothalamic subdivisions across PHy and THy, different nuclear structures originate within each of these two domains, and also why singular structures arise at the ATD, e.g., the suprachiasmatic nuclei, the arcuate nucleus, the median eminence and the neurohypophysis.

## Introduction

The developing mouse hypothalamus has been extensively investigated as regards its genoarchitecture in recent years (Shimogori et al., 2010; Diez-Roux et al., 2011; Puelles et al., 2012). Many results corroborated a fundamental organization in terms of a dorsoventral array of longitudinal zones showing characteristic molecular profiles. Thus, the alar hypothalamus appears divided dorsoventrally into the paraventricular and subparaventricular areas, and the basal hypothalamus likewise displays dorsoventrally arranged tuberal and mamillary areas (Morales-Delgado et al., 2011, 2014; Puelles et al., 2012; Díaz et al., 2015, this Issue). Each of these primary longitudinal domains can be subdivided into thinner longitudinal subdomains, or microzones (Puelles, 2013). The pattern bespeaks of an important role of dorsoventral patterning in the anatomical structure of the hypothalamus. It allows us to explain similarities in differentiation patterns (and eventually functions) shared along these histogenetically characteristic regions [e.g., precocious neurogenesis along a dorsal tuberal microzone (the classic hypothalamic cell cord), distribution of hypophysiotropic cell populations along the paraventricular area, the continuum formed by the dorsomedial and arcuate nuclei, origin of histaminergic neurons along the ventral tuberal microzone, and the distribution of *Otp*-expressing neurons within the mamillary region; the adult arrangement of glutamatergic and GABAergic cell populations also relates to the same dorsoventral pattern of longitudinal zones (Puelles et al., 2012)].

**Figure 1:**
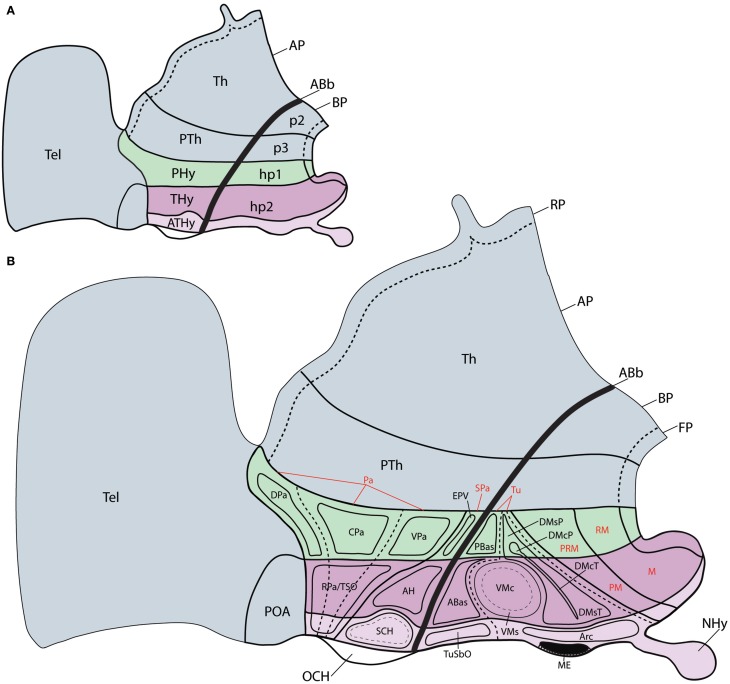
**Schemata representing the updated prosomeric model of the forebrain (excepting diencephalic p1), in which the general position, morphological organization and principal nuclear and genoarchitectonic subdivisions of the hypothalamus are detailed. (A)** Schema showing the two rostral diencephalic prosomeres (p2 and p3) and the hypothalamo-telencephalic prosomeres 1 and 2 (hp1 and hp2). Note hp1 contains the peduncular hypothalamic region (PHy), whereas hp2 includes the terminal hypothalamic region (THy) and the rostralmost, median acroterminal domain (ATHy). **(B)** Schema of the main hypothalamic progenitor areas distributed across the dorsoventral and anteroposterior dimensions. The longitudinal alar/basal boundary (ABb) is indicated as a thick dark line. The hypothalamic area is subdivided rostrocaudally into neuromeric THy and PHy parts (pink and green, respectively). Alar territories (AP) are shown on the left, and basal ones on the right. The alar hypothalamus is subdivided dorsoventrally into paraventricular (TPa/PPa) and subparaventricular (TSPa, PsPa) areas, plus the corresponding acroterminal domains. The paraventricular area shows a general tripartition into dorsal, central and ventral subdivisions (DPa, CPa, VPa). The basal hypothalamus is also subdivided dorsoventrally into the large tuberal/retrotuberal (Tu/RTu) area and the primary mamillary/retromamillary (M/RM) area, plus the corresponding acroterminal regions. The THy/PHy parts of the hypothalamic floor lie underneath (FP). Moreover, the Tu/RTu region is subdivided into three dorsoventral parts: TuD/RTuD, TuI/RTuI, and TuV/RTuV, and the primary M/RM area is subdivided into perimamillary/periretromamillary area (PM/PRM) and the secondary M/RM area. Some well-known nuclear elements of the hypothalamus are represented within their respective topography relative to the molecular domains; note some of these positions are postmigratory (see the list for abbreviations).

However, conventional analysis of nuclear structure in the hypothalamus already underlines the existence of grisea that apparently only develop within the rostral or caudal moieties of the cited longitudinal bands. Clearcut examples of this are the anterolateral, suprachiasmatic, anterior, ventromedial, ventral/dorsal premamillary and arcuate nuclei, only present rostrally, and the main paraventricular nucleus, jointly with the entopeduncular nuclei and the subthalamic nucleus, only present caudally. Moreover, distinct mamillary (rostral) and retromamillary (caudal) formations have been distinguished within the mamillary region. Such differences already suggested an anteroposterior bipartition of the hypothalamus (Puelles and Rubenstein, 2003; Puelles et al., 2012) finally postulated that such bipartition responded to the existence of two distinct neuromeric fields stretching through the hypothalamus and telencephalon continuum (hypothalamic prosomeres hp1 and hp2, named in caudorostral order) (Figure 1A). The corresponding hypothalamic parts were identified as *peduncular* and *terminal* hypothalamus (PHy, THy; note there is a rough correspondence of these *transverse* parts with historical use by Herrick, 1910 and others of the notion of “dorsal” and “ventral” hypothalamus, of course referring to a completely different length axis; this use has been abandoned by columnar authors in recent times, e.g., see Swanson, 1992, 2003; other authors identified THy as “hypothalamus” and PHy as “subthalamus,” e.g., Reinoso-Suárez, 1960; Richter, 1965; see discussion in Puelles et al., 2012).

In any case, these antecedents suggest that, apart of longitudinal genoarchitectonic patterns, there is also room for anteroposterior molecular differences across PHy and THy (that is, hp1 and hp2), without forgetting the existence of a singular rostromedian transverse territory within THy, recently identified as the acroterminal domain (ATD; Puelles et al., 2012), where specialized formations are characteristic (Figures 1A,B). One expects some differential molecular pattern across adjacent neuromeres that share other determinants, particularly so when the respective adult derivatives are anatomically diverse. In that case, the crucial expected observation is that various partial zonal or microzonal patterns restricted to each of the units show a common transverse boundary that agrees with the postulated interneuromeric border. This corresponds mainly to the intrahypothalamic boundary in our field of interest, but also, secondarily, to the acroterminal vs. terminal boundary.

We already had partial knowledge of some such molecular anteroposterior differences (Shimogori et al., 2010; Puelles et al., 2012), but we resolved to perform a wider data-mining study within the Allen Developing Mouse Brain Atlas database (developingmouse.brain-map.org), whose results we report here. We searched for gene markers expressed at early stages (E11.5, 13.5) whose expression domain either at the ventricular or mantle zones was significantly restricted to the terminal or peduncular moiety of one or several longitudinal zones. Interesting candidates were also examined at older stages, to assess the correlation of specific nuclei to the molecular pattern.

## Materials and methods

We report here on 37 gene expression patterns (Table 1), analyzed from *in situ* hybridization images downloaded from the *Allen Developing Mouse Brain Atlas*.^1^ These are mostly sagittal sections; while this section plane is appropriate for the analysis of spatial dorsoventral and anteroposterior topographic relationships needed in our analysis, the visualization of some anatomic landmarks may be compromised. We recurred to careful analysis of all sagittal (eventually also coronal) section planes shown at the *Allen Atlas*, as well as to our extensive experience with multiple planes of sections through the mouse hypothalamus. We correlated the positions of labeled cells at the time points available at the *Allen Atlas* (embryonic days E11.5, E13.5, E15.5, and E18.5, and postnatal day P4) with the genoarchitectonically distinct areas and conventional nuclei, following the model of Puelles et al. (2012).

**Table 1:** **37 genes analyzed, classified according to their mouse genome informatics ID, gene family classification (HUGO) and functional gene ontology (GO)**.

**Gene**	**MGI: ID**	**Gene family (HUGO nomenclature committee)**	**Gene ontology (GO): key function**
Ascl1	96919	Basic helix-loop-helix proteins	bHLH transcription factor binding
Cadm1	1889272	Immunoglobulin superfamily	Cell adhesión
Calb1	88248	EF-hand domain containing	Calcium ion binding
Erbb4	104771		ATP binding
Fgf8	99604	Endogenous ligands	Fibroblast growth factor receptor binding, chemoattractant activity
Fgf10	1099809		Fibroblast growth factor receptor binding, chemoattractant activity
Fgf15	1096383		Fibroblast growth factor receptor binding, growth factor activity
Fgf18	1277980		Fibroblast growth factor receptor binding, growth factor activity
FoxB2	1347468	Forkhead boxes	DNA binding
Foxp1	1914004	Forkhead boxes	DNA binding
Gsc	95841	Homeoboxes/PRD class	DNA binding
Irx1	1197515	Homeoboxes/TALE class	DNA binding
Irx3	1197522	Homeoboxes/TALE class	DNA binding
Irx5	1859086	Homeoboxes/TALE class	DNA binding
Lef1	96770		Beta-catenin binding
Lmo4	109360		DNA binding
Meis2	108564	Homeoboxes/TALE class	DNA binding
Mfap4	1342276	Fibrinogen C domain containing	Cell adhesión
Nkx6.2	1352738	Homeoboxes/ANTP class:NKL subclass	DNA binding
Nr5a1	1346833	Nuclear hormone receptors	DNA binding
Otp	99835	Homeoboxes/PRD class	DNA binding
Otx1	97450	Homeoboxes/PRD class	DNA binding
Otx2	97451	Homeoboxes/PRD class	DNA binding
Plagl1	1100874	Zinc fingers, C2H2-type	DNA binding
Pmch	97629	Endogenous ligand	Homone activity
Pomc	97742	Endogenous ligands	Homone activity
Prdm12	2685844	Zinc fingers, C2H2-type	1-hydroxypyrene methyltransferase activity
Rax	109632	Homeoboxes/PRD class	DNA binding
Rgs4	108409	Regulation of G-protein signaling	GTPase activator activity
Rprm	1915124		Molecular function
Sall3	109295	Zinc fingers, C2H2-type	DNA binding
Satb2	2679336	Homeoboxes/CUT class	DNA binding
Six3	102764	Homeoboxes/SINE class	DNA binding
Six6	1341840	Homeoboxes/SINE class	DNA binding
Tbr1	107404	T-boxes	DNA binding
Tcf7l2	1202879		Beta-catenin binding
Vax1	1277163	Homeoboxes/ANTP class:NKL subclass	DNA binding
Zic1	106683	Zinc fingers, C2H2-type	DNA binding
Zic5	1929518	Zinc fingers, C2H2-type	DNA binding

## Results

In this section we will describe selected examples that best represent expression restricted to either PHy or THy, or to the ATD.

### Acroterminal patterns

We deal first with observations at the ATD, that is, with genes restricted in expression to the ATD, or combining ATD and THy patterns.

**Figure 2:**
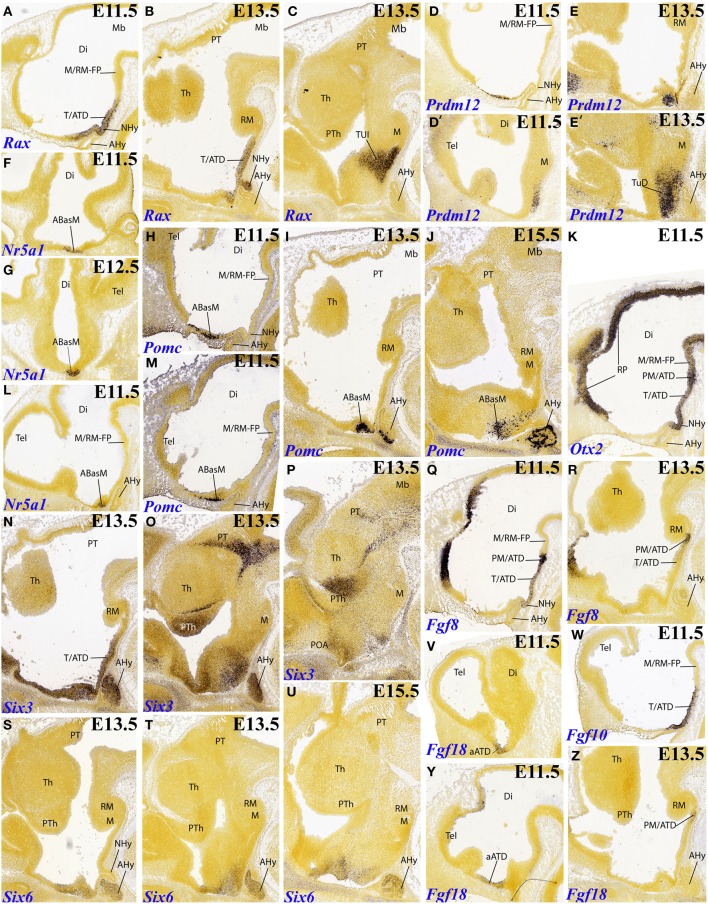
**Sagittal, parasagittal (A,B–E,H–Z) and horizontal (F,G) sections through the mouse secondary prosencephalon and diencephalon at E11.5, E13.5, and E15.5, showing relevant examples of selective hypothalamic gene expression at the acroterminal domain (ATD) and terminal territory: *Rax* (A–C), *Prdm12* (D–E′), *Nr5a1* (F,G,L), *Pomc* (H–J,M), *Otx2* (K), *Six3* (N–P), *Fgf8* (Q,R), *Six6* (S–U), *Fgf18* (V,Y,Z), and *Fgf10* (W)**. All images were downloaded from the Allen Developing Mouse Brain Atlas (http://developingmouse.brain-map.org/). For abbreviations, see the list.

The gene *Rax*, for instance, appears selectively expressed at E11.5 and E13.5 in ventricular cells along the tuberal sector of the ATD; the neurohypophysis primordium lies centered within this domain, and is also positive (Figures 2A,B, **6B**). It is unclear whether this tuberal domain includes the complete TuD subdomain, or rather only its ventralmost part (see Figure 2B; compare other patterns shown in Figures 2E,I). In contrast, the perimamillary and mamillary parts of the ATD remain free of label, as does the whole alar ATD. At E13.5, some *Rax* signal also appears spread out into the adjacent terminal tuberal territory (TuI; Figure 2C), but was not observed to reach any part of the PHy.

The gene *Prdm12* is expressed already at E11.5 at the dorsal tuberal part of THy (TuD), restricted to the mantle zone, and clearly encompassing the corresponding median ATD territory (Figures 2D,D′). Two days later, this pattern is amplified throughout the anterobasal derivatives of TuD (Figures 2E,E′), and additional signal appears more laterally, distributed along the alar subparaventricular band (both THy and PHy; not shown).

At E11.5 and E12.5, there is expression of *Nr5a1* that is clearly restricted to the TuD sector of the ATD (TuD/ATD, or ABasM area; Figures 2F,G,L). At later stages this marker also appears within the adjoining TuD zone of THy (TuD/THy, or ABas area; **Figures 4H,J**, **6F**); Puelles et al. (2012) commented on the relationship of these TuD-originated primordia in the development of the migrated ventromedial nucleus.

*Pomc* was first described in an early median population by Shimogori et al. (2010); these cells apparently migrate subsequently into the arcuate nucleus area (TuI/ATD), as was corroborated by Puelles et al. (2012). The latter authors ascribed the initial *Pomc*-positive locus to the ABasM (the TuD/ATD sector). We examined this pattern again at E11.5, confirming positive mantle cells within ABasM, but accompanied by more dorsal cells within the overlying alar sectors of the ATD; the signal seemed restricted to the ventricular zone at these alar loci (Figures 2H,M); it was no longer present at E13.5, when *Pomc* cells densely aggregate at the ABasM mantle (Figure 2I). This result opens the possibility that the real origin of *Pomc* cells lies in the alar ATD. Long dorsoventral tangential migrations of peptidergic hypothalamic neurons are by no means rare (see Díaz et al., 2015; this Issue). At E15.5 the ABasM population appears spread out dorsoventrally, consistently with its postulated final migration into the arcuate region (Figures 2J, **6B**).

*Otx2* appears expressed selectively along the mamillary and tuberal sectors of the ATD up to and including the neurohypophysis primordium at E11.5; interestingly, the selected midsagittal section also shows widespread labeling of the whole roof plate, all the way to its end at the anterior commissure locus, as well as of the whole floor plate, all the way to the mamillary region; this causes here continuity of floor- and ATD-related signal (Figures 2K, **6D**). At E13.5 this ATD expression of *Otx2* is much reduced, but some signal still remains at the neurohypophysis and at the perimamillary sector of the ATD, next to novel signal appeared within the mamillary area of THy (**Figure 5N**).

*Six3*, a gene already expressed in the rostral neural plate, is well-known to become restricted subsequently as a marker of the rostromedian telencephalon and the alar and basal hypothalamus, but excluding the mamillary region (Puelles et al., 2012). This ATD-related distribution is shown here at E13.5 (Figures 2N, **6E**); more lateral sections reveal that *Six3* signal also spreads into adjacent alar and tuberal basal parts of THy, but not into PHy (Figures 2O,P, **6E**). Curiously, irrespective of this restriction of its neural tube expression domain along both DV and AP dimensions, the whole hypothalamus disappears in the null mutation (Lagutin et al., 2003). As nuclear structure develops, *Six3* signal appears mainly at the suprachiasmatic nucleus (one of the ATD derivatives; not shown).

On the other hand, *Six6* appears more selectively expressed at the alar ATD, as well as at the TuD sector of the basal ATD, at E11.5 (weak; not shown) and E13.5 (Figures 2S,T, **6A**). The pattern is unchanged at E15.5, though the signal seems to predominate in the ATD regions of the alar paraventricular and subparaventricular areas (Figure 2U).

We examined all *Fgf* genes, and found some relevant aspects. At E11.5 *Fgf8* is expressed strongly at the perimamillary ATD sector, and more weakly along the tuberal ATD sector, just including the neurohypophysis anlage (Figures 2Q, **6C**). However, this pattern appears much reduced at E13.5, with signal remaining only (weakly) at the PM/ATD (Figure 2R). Note this small sector also appears labeled at E13.5 with *Otx1* (**Figure 5M**), *Otx2* (**Figure 5N**) and *Cadm1* (**Figure 5R**). *Fgf10* roughly reproduces the initial tuberal ATD expression domain of *Fgf8*, with stronger signal, though the PM/ATD sector is in this case inconspicuous (Figures 2W, **6C**); at later stages the *Fgf10* signal becomes restricted to the neurohypophysis (not shown). Finally, *Fgf18* transiently appeared expressed at E11.5 within the alar ATD region (Figures 2V,Y, **6A**); but at E13.5 a weak signal is observed at the PM/ATD (Figure 2Z).

The gene *Nkx6.2* was found expressed only bilaterally at the ABas area (TuD/THy) at E11.5 (**Figures 4F**, **7A**), but no signal was detected in two intervening sections depicting the midsagittal plane (not shown). We conclude from this that the ATD is specifically excluded from this expression pattern.

A further gene pattern highlighting the ATD is *Sall3*. This pattern covers at E13.5 essentially the whole basal ATD, starting with rather weak signal at its TuD sector. The expression is weakest at the neurohypophysis anlage and then becomes strong ventral to it, along the TuI, TuV (TM) and mamillary sectors (**Figure 5P**). This gene is also expressed within ventricular and mantle zone of the mamillary area of THy, but distinctly not at the mantle zone of the retromamillary area of PHy (data not shown).

### Terminal hypothalamus patterns

**Figure 3:**
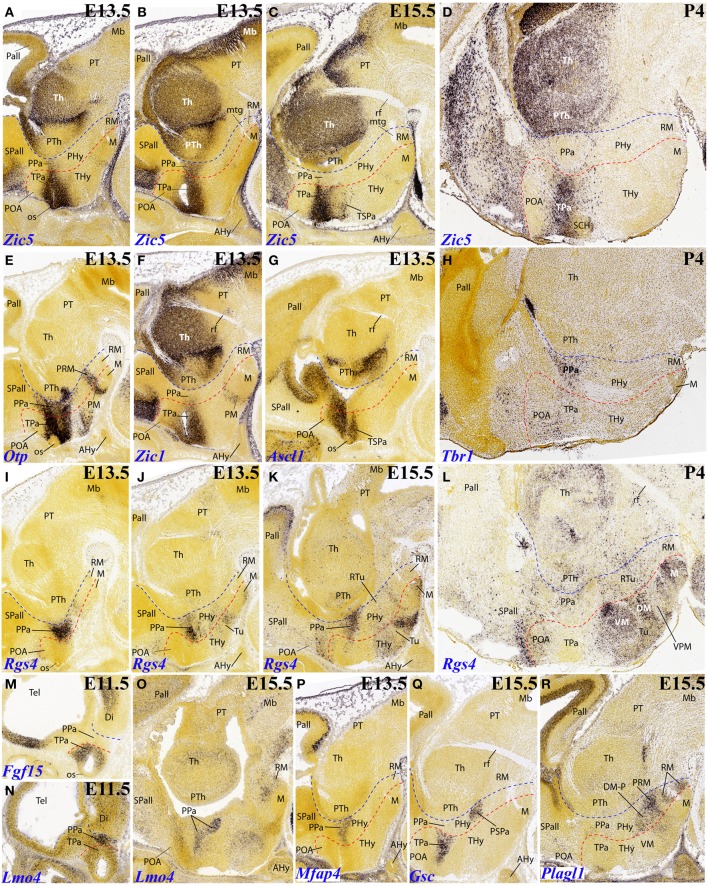
**Sagittal and parasagittal (A–R) sections through the mouse secondary prosencephalon and diencephalon at E11.5, E13.5, E15.5, and one P4 image, showing relevant examples of hypothalamic genes expressed selectively at the terminal (THy) and peduncular (PHy) territories: *Zic5* (A–D), *Otp* (E), *Zic1* (F), *Ascl1* (G), *Tbr1* (H), *Rgs4* (I–L), *Fgf15* (M), *Lmo4* (N,O), *Mfap4* (P), *Gsc* (Q) and *Plagl1* (R)**. All images were downloaded from the Allen Developing Mouse Brain Atlas (http://developingmouse.brain-map.org/). For abbreviations, see the list. Red dotted line: THy/PHy boundary. Blue dotted line: PHy/p3 boundary.

*Zic5*, belonging to the *Zic* gene family related to the specification of alar neural regions, shows an interesting dynamic expression pattern. At E13.5 its signal appears restricted to the ventricular zone of the alar paraventricular area across both THy and PHy (extending into the eye stalk, which is an ATD component). However, this expression is distinctly weaker at PHy than at THy (Figures 3A,B). At E15,5, the paraventricular mantle zone shows strong expression (some cells also disperse into the subjacent subparaventricular area), but only at the THy moiety (Figures 3C, **6F**). This situation clearly remains unchanged at P4 (Figure 3D; note the blank main paraventricular nucleus and the negative suprachiasmatic nucleus. For comparison we inserted the pattern of *Otp*, which shows an equally dense labeled mantle in THy and PHy (Figure 3E), and the pattern of *Tbr1* at P4, which emphasizes the PHy derivative of the paraventricular area (Figure 3H, to compare with Figure 3D).

*Zic1* also seems predominantly expressed in the terminal paraventricular area (Figure 3F).

*Ascl1* is selectively expressed at E13.5 within the terminal subparaventricular area mantle (Figure 3G).

*Rgs4* seems first restricted to the peduncular paraventricular area at E13.5 (Figures 3I,J), though additional signal appears within the terminal basal territory. Various positive basal populations of THy are distinguished at E15.5 (Figures 3K, **7C**). Practically the whole terminal tuberal and mamillary regions show *Rgs4* signal at P4, contrasting with only few and dispersed positive cells within the basal PHy (Figure 3L; note in particular that the ventral premamillary nucleus, a migrated derivative of the retromamillary area according to Puelles et al., 2012, appears completely negative within the tuberal zone).

**Figure 4:**
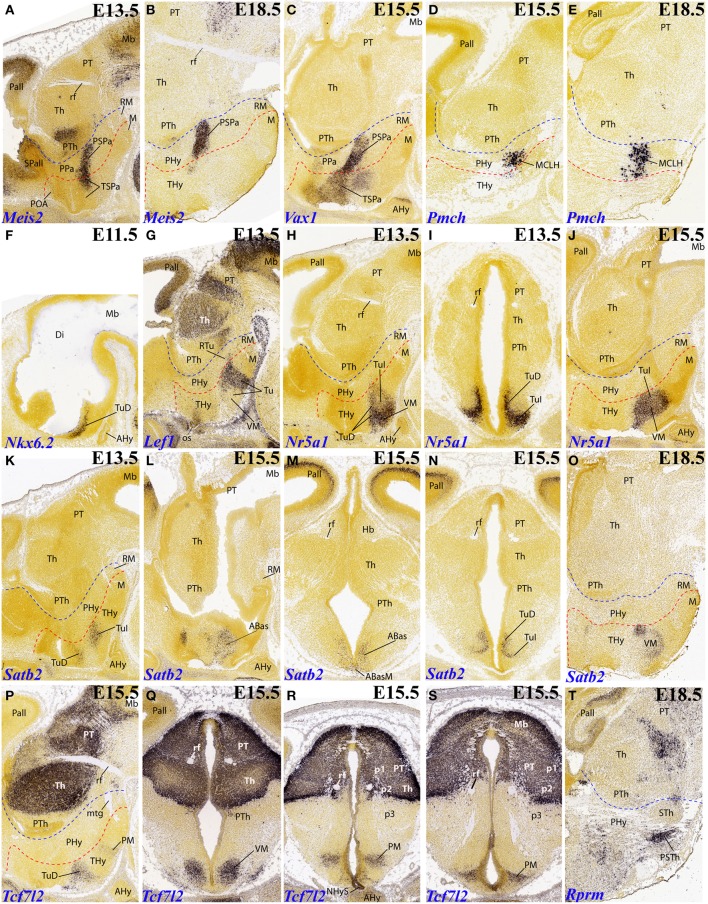
**Sagittal, parasagittal (A–H,J–L,O,P,T) and transversal (I,M,N,Q–S) sections through the mouse secondary prosencephalon and diencephalon at E11.5, E13.5, E15.5, and E18.5, showing relevant examples of the hypothalamic genes expressed selectively at the terminal (THy), peduncular (PHy) or acroterminal (ATD) territories: *Meis2* (A,B), *Vax1* (C), *Pmch* (D,E), *Nkx6.2* (F), *Lef1* (G), *Nr5a1* (H–J), *Satb2* (K–O), *Tcf7l2* (P–S), and *Rprm* (T)**. All images were downloaded from the Allen Developing Mouse Brain Atlas (http://developingmouse.brain-map.org/). For abbreviations, see the list. Red dotted line: THy/PHy boundary. Blue dotted line: PHy/p3 boundary.

We found expression of *Fgf15* restricted at E11.5 to the terminal alar hypothalamus, though leaving the acroterminal optic stalk negative (Figure 3M). *Gsc* expression was observed selectively in the terminal paraventricular zone at E13.5 and E15.5 (Figure 3Q). The terminal pattern of *Nkx6.2* was described above (Figure 4F).

It is unclear whether *Lef1* expression in the tuberal hypothalamus shows a difference between THy labeling (which seems extensive) and PHy labeling (which seems minoritary, restricted to a small tail-like area; Figure 4G). Note no signal corresponds to the ventromedial nucleus, possibly due to the ectopic migrated nature of this tuberal mass, originated at the overlying TuD domain (Puelles et al., 2012).

The VM primordium can be selectively labeled with the *Nr5a1* marker, as advanced above (Figures 4H–J, **6F**); note the cross-section shows the migratory course, leading TuD–originated cells into TuI. A similar labeling pattern restricted to THy is provided by *Satb2*, whose initial expression is associated to TuD (ABas/THy and ABasM/ATD) (Figures 4K–N) and later the cells translocate analogously to *Nr5a1*–expressing cells into the definitive VM nucleus, particularly into its rounded dorsomedial subnucleus (Figure 4O). Another gene marker associated to the VM is *Tcf7l2*, a massive marker of thalamus and pretectum, which in addition already shows at E15.5 label at the TuD area of THy and ATD, from where labeled cells translocate subsequently into the migrated VM (Figures 4P,Q).

**Figure 5:**
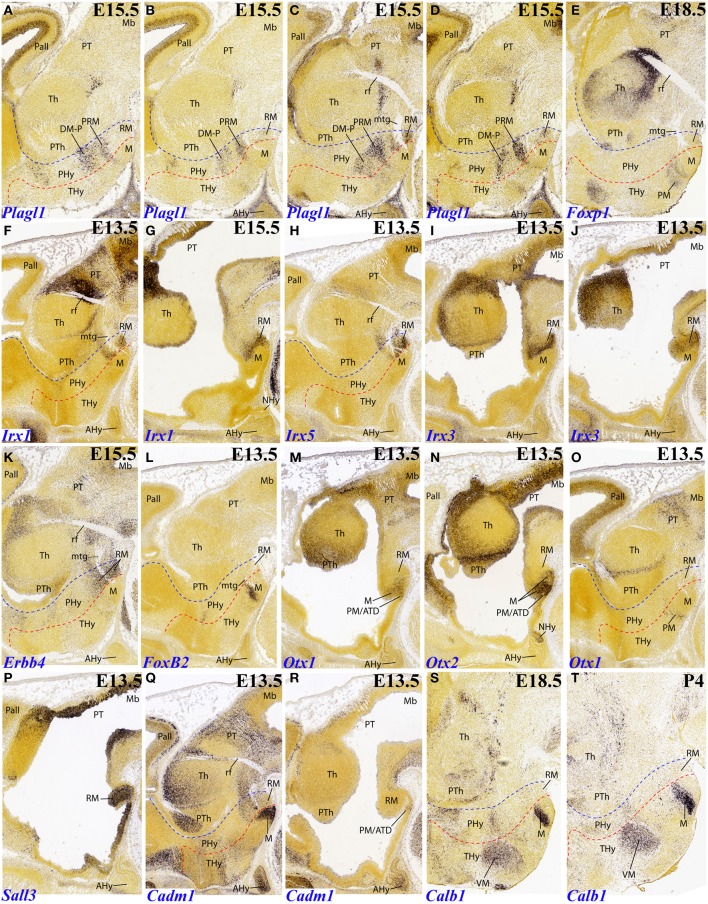
**Sagittal and parasagittal (A–T) sections through the mouse secondary prosencephalon and diencephalon at E11.5, E13.5, E15.5, E18.5, and P4, showing relevant examples of hypothalamic genes expressed selectively at the terminal (THy), peduncular (PHy) or acroterminal (ATD) territories: *Plagl1* (A–D), *Foxp1* (E), *Irx1* (F,G), *Irx5* (H), *Irx3* (I,J), *Erbb4* (K), *FoxB2* (L), *Otx1* (M,O), *Otx2* (N), *Sall3* (P), *Cadm1* (Q,R), and *Calb1* (S,T)**. All images were downloaded from the Allen Developing Mouse Brain Atlas (http://developingmouse.brain-map.org/). For abbreviations, see the list. Red dotted line: THy/PHy boundary. Blue dotted line: PHy/p3 boundary.

In addition, *Tcf7l2* also labels selectively the hypophyseal stalk within ATD, as well as the perimamillary band within THy (Figures 4R,S). The perimamillary THy band was also selectively labeled by other markers, such as *Zic1* (Figure 3F), *Foxp1* (Figure 5E) and *Otx1* (Figures 5O, **7B**).

A surprising finding was the expression of *Rprm* at the parasubthalamic nucleus, while the subthalamic nucleus itself remains wholly devoid of this signal (Figure 4T). It had been previously supposed that the PSTh originated jointly with the STh at the peduncular retromamillary area (Puelles et al., 2012). The present result suggests that perhaps the PSTh is terminal in origin, or at least comes from a different origin than the STh nucleus.

Several genes show expression restricted to the terminal mamillary body complex, leaving the retromamillary area negative. However, these patterns may show restriction within the mamillary area itself, sometimes to quite small cell groups difficult to interprete. One mystifying cell group appears labeled selectively by *FoxB2* at E13.5 (Figures 5L, **7F**). It parallels the origin of the mamillotegmental tract and apparently represents only a minor part of the medial mamillary nucleus. *Otx1* labels faintly the mamillary ventricular zone (Figure 5M), while *Otx2* also labels the mantle zone (Figure 5N). *Cadm1* only labels a compact superficial population visible at relatively lateral section levels, possibly related to the lateral mamillary nucleus (Figure 5Q). Finally, *Calb1* is also expressed in a subpopulation of the mamillary body which lies deep to the brain surface at intermediate section levels (Figures 5S,T); note there is also *Calb1* expression restricted to the VM nucleus.

### Peduncular hypothalamus patterns

As already mentioned, at early stages *Rgs4* shows restricted labeling of the peduncular component of the paraventricular area. At E13.5 this seems to imply the whole dorsoventral extent of the main paraventricular nucleus (Figures 3I,J), though at E15.5 the expression seems restricted to its ventral component (Figures 3K, **7C**).

*Lmo4* and *Mfap4* likewise appear expressed at the peduncular paraventricular area, predominantly at its central and ventral subdivisions (Figures 3N–P, **7E**).

**Figure 6:**
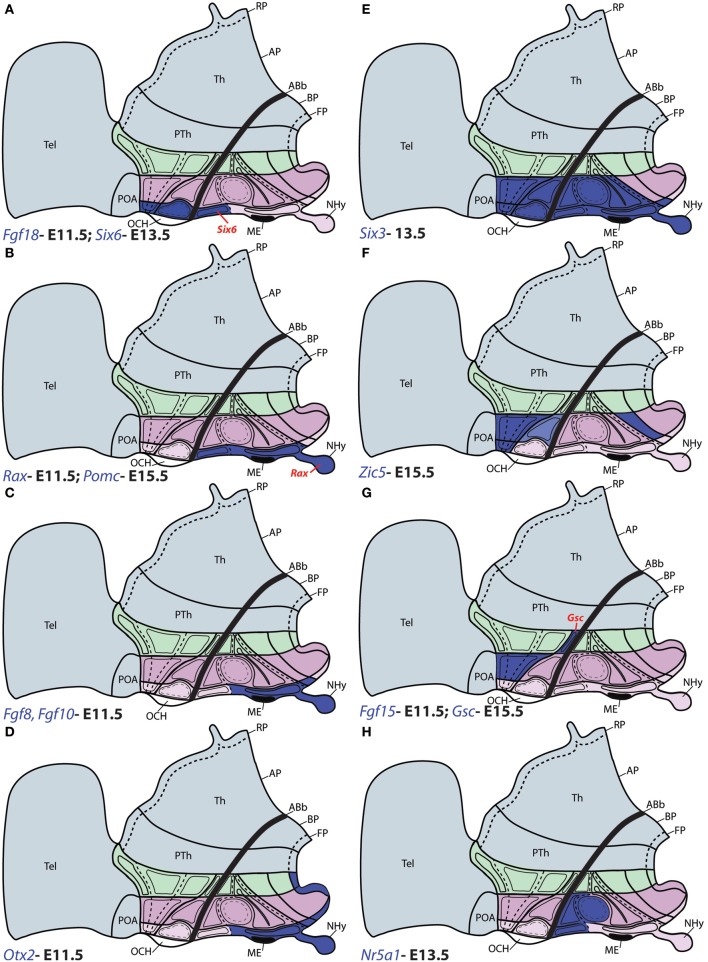
**Schematic maps of characteristic genoarchitectonic patterns in the hypothalamus, illustrating studied patterns selective for the acroterminal (ATHy), terminal (THy) or peduncular (PHy) hypothalamic domains**. Compare subdivisions with Figure 1. **(A)** The *Fgf18* and *Six6* domains overlap within alar ATHy domains; but only *Six6* (red tag) is detected at the acroterminal TuD domain. **(B)** *Rax* and *Pomc* were detected along the acroterminal tuberal domains; but only *Rax* was observed at the NHy (red tag). **(C)** *Fgf8* and *Fgf10* were detected along the intermediate and ventral basal tuberal acroterminal domains, including NHy, extending also into the acroterminal perimamillary area. **(D)** *Otx2* was observed at the acroterminal TuI, TuV, PM, and M domains, as well as along the THy and PHy floor plate. **(E)** *Six3* was throughout the alar domains of ATHy and THy; but at the basal plate its expression was restricted to the Tu region of ATHy and THy, and ATHy of the PM domain. **(F)** *Zic5* expression was detected at the alar TPa (and corresponding ATHy area) and TSPa domains of THy (but respecting the local acroterminal suprachiasmatic nucleus). Additionally, *Zic5* signal also appeared restricted to the PM region of THy. **(G)** *Fgf15* and *Gsc* were detected jointly at the TPa area; but only *Gsc* was detected in the PSPa domain (red tag). **(H)** *Nr5a1* expression was detected in the TuD domain across ATHy and THY, and the migrated derivatives of these areas entering the ventromedial nucleus also expressed this gene within TuI.

**Figure 7:**
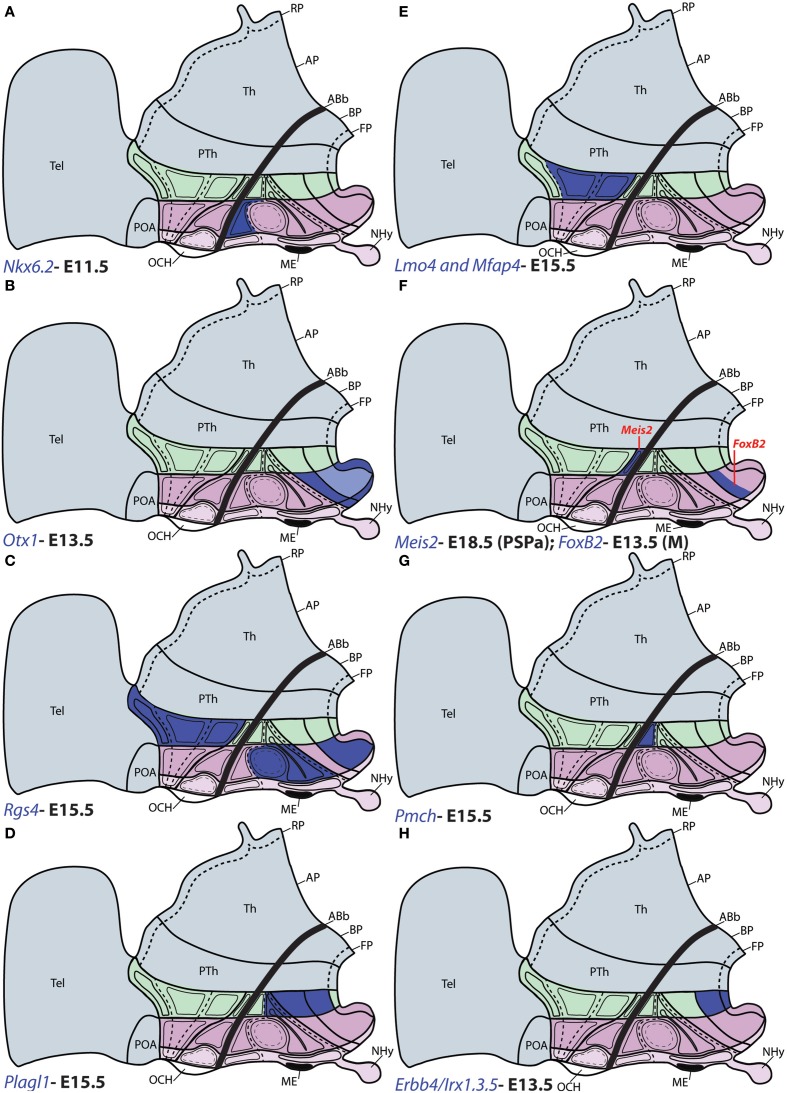
**Schematic maps of characteristic genoarchitectonic patterns in the hypothalamus, illustrating studied patterns selective for the terminal (THy) or peduncular (PHy) hypothalamic domains**. Compare subdivisions with Figure 1. **(A)** *Nkx6.2* was detected in the TuD domain of THy. **(B)** *Otx1* expression was observed in the PM, M, and FP areas across ATHy and THy. **(C)** *Rgs4* was detected at the basal TuI, TuV, and M areas from THy, as well as throughout the Pa area from PHy. **(D)** *Plagl1* was detected at the basal RTuI, RTuV, PRM, and RM from PHy. **(E)** *Lmo4* and *Mfap4* were expressed selectively at the CPa and VPa paraventricular subdivisions within alar PHy. **(F)** *Meis2* signal (red tag) was detected at the small PSPa domain; *FoxB2* (red tag) was observed in a previously unknown dorsal subdivision of M domain (THy). **(G)** *Pmch* signal was detected only at the RTuD domain from basal PHy. **(H)** *Erbb4, Irx1, Irx3*, and *Irx5* were detected at the RM and PHy FP.

*Gsc* appears expressed selectively at E15.5 within the peduncular portion of the subparaventricular area, apart separate expression in the terminal paraventricular area is observed (Figures 3Q, 6G). Another marker distinguishing this PHy subparaventricular domain is *Meis2*. At E13.5 its expression seems to extend partly into the THy (Figure 4A), but at E18.5 the signal is limited strictly to PHy (Figures 4B, 7F). Moreover, *Vax1* shows at E15.5 differential subparaventricular labeling, with weaker and more disperse signal within the THy (suprachiasmatic and anterior hypothalamic nuclei), including some ventrally migrated cells in the underlying tuberal area, and more compact signal within the PHy component (Figure 4C).

*Plagl1* expression is restricted at E15.5 to the basal PHy, involving the retromamillary area (RM), the periretromamillary area (PRM) and the peduncular dorsomedial nucleus (DM-P, belonging to the RTu area), but not the posterobasal area (Figures 3R, 5A–D, 7D).

The precociously differentiating superficial basal cell group known as the magnocellular lateral hypothalamic nucleus (MCLH), a derivative of the posterobasal area (the PHy component of the RTuD area) appears selectively marked with *Pmch*, as shown here at E15.5 and E18.5 (Figures 4D,E, 7G).

Several genes show restricted expression at the retromamillary area. For instance, *Irx1* (Figures 5F,G, 7H), *Irx5* (Figures 5H, 7H), *Irx3* (Figures 5I,J, 7H), *Erbb4* (Figures 5K, 7H), and *Sall3* (Figure 5P; compare with Figure 5J).

## Discussion

Our results clearly illustrate that, as expected, the acroterminal, terminal, and peduncular subregions of all the shared dorsoventral domains are further differentially specified by a number of genes. These molecular subdivisions probably underpin causally the development of particular nuclei within these areas. Most of the genes studied code DNA-binding proteins, which probably are involved in fate and identity specification. These observations in general agree with additional data reported by Shimogori et al. (2010), Diez-Roux et al. (2011) and Puelles et al. (2012).

While in most cases the observed restricted expression domains were limited to a single longitudinal zone, in the cases of *Rgs4* and *Plagl1* a large part of the basal plate area, including the TuI/RTuI, PM/PRM, and M/RM domains was neatly divided into THy and PHy moieties. In all cases the labeled domains stopped at the postulated intrahypothalamic boundary, corroborating its role as a general interneuromeric boundary.

Remarkably, the acroterminal domain appeared also well delimited molecularly from the remaining part of THy, so that we can speak now of a distinct acro-terminal border. This domain also showed a diversity of molecular profiles along its dorsoventral dimension, illustrating that its different sectors producing characteristic derived structures are also differentially specified by the antagonism of dorsalizing and ventralizing patterning mechanisms.

Most of the gene expression patterns analyzed are consistent with neuroepithelial compartments previously delimited in the prosomeric model (Puelles et al., 2012); in contrast, these expression patterns cannot be explained or classified on the basis of columnar ideas, since this alternative model was not developed to the point of postulating such fine subdivisions. From the viewpoint of the updated prosomeric model these results demonstrate that the postulate of two hypothalamo-telencephalic prosomeres (hp1, hp2), which cut transversely across the set of five main longitudinal dorsoventral zones previously distinguished in the hypothalamus (Figure 1), dividing each of them in two molecularly distinct anteroposterior domains, satisfies the assumption that differential gene expression patterns should characterize each of these prosomeres, irrespective that they evidently share the fundamental dorsoventral molecular regionalization of the hypothalamus.

The supposed evolutionary advantage of neuromeres lies precisely in their capacity to maintain a common organization of shared properties along the dorsoventral dimension of a series of developmental units (metamerism), while varying subtly the molecular profile (molecular identity) of each neurogenetic unit along the anteroposterior axis, that is, varying the sorts of neurons produced within similar environments. This allows generic properties, such as the capacity to guide the longitudinal growth of the optic tract, to be combined with the differential development of specific target areas for this tract within given prosomeres; this multiplies with a minimum of genetic instructions the sorts of operational algorithms that the brain can perform on any given afferent signal.

We also found some unexpected data, representing molecularly distinct mantle subdivisions that are smaller than the partitions contemplated so far in the updated prosomeric model (e.g., *FoxB2* expression). These results suggest that there may be grounds to further analyze morphologically and developmentally the regions of the model where this occurs, an endeavor that may lead eventually to a more advanced and comprehensive version of the model.

The specialized median acroterminal domain postulated by Puelles et al. (2012) was a logical necessity, since the differential structures observable in this domain, both as regards the dorsoventral dimension (roof, alar, basal, and floor sectors) and with respect to the caudally adjacent structural elements of the THy, only could be conceived if the underlying causal conditions, including molecular ones, are likewise differential. Though some evidence regarding our prediction of differential gene markers within the ATD was adduced already by Puelles et al. (2012), recollected from earlier reports cited therein, it was conceptually important to fill all the remaining theoretical pigeonholes with some corresponding singular markers. This aim was satisfactorily fulfilled with our present analysis of available data in the Allen Developmental Mouse Brain Atlas database, though many other selective markers probably will appear in the future. Traditionally it had been habitual to look away from this area in gene mapping studies, probably because it seemed to behave differently from the basic structural schema of the hypothalamus found in textbooks. The acroterminal concept and the presently available evidence of its distinct molecular signature, simultaneously immersed in the general molecular pattern of the hypothalamus, hopefully will attract attention to it and to its particular mode of morphogenesis.

### Conflict of interest statement

The authors declare that the research was conducted in the absence of any commercial or financial relationships that could be construed as a potential conflict of interest.

## Footnotes

^1^http://developingmouse.brain-map.org/

## Abbreviations

aATD: Alar acroterminal domain
ABas: Anterobasal nucleus
ABasM: Median acroterminal ABas
ABb: Alar basal boundary
AH: Anterior hypothalamic nucleus
AHy: Adenohyphophysis
AP: Alar plate
Arc: Arcuate nucleus
ATHy: Acroterminal hypothalamus
BP: Basal plate
CPa: Central portion of the peduncular paraventricular area
Di: Diencephalon
DM: Dorsomedial hypothalamic nucleus
DM-P: Dorsomedial hypothalamic nucleus—Peduncular part
DMcP: Core portion of the peduncular DM
DMcT: Core portion of the terminal DM
DMsP: Shell portion of the peduncular DM
DMsT: Shell portion of the terminal DM
DPa: Dorsal portion of the peduncular paraventricular area
EPV: Ventral entopeduncular nucleus
FP: Floor plate
Hb: Habenula
hp1: Hypothalamo-telencephalic prosomere 1
hp2: Hypothalamo-telencephalic prosomere 2
M: Mamillary region
M/RM-FP: Mamillary/retromamillary floor plate
Mb: Midbrain
MCLH: Magnocellular lateral hypothalamic nucleus
ME: Median eminence
mtg: Mamillotegmental tract
NHy: Neurohypophysis
NHyS: Neurohypophyseal salk
OCH: Optic chiasm
os: Optic stalk
Pa: Parventricular hypothalamic complex
Pall: Pallium
PBas: Posterobasal area and nucleus
PHy: Peduncular hypothalamus
PM: Perimamillary region
PM/ATD: Acroterminal part of perimamillary region
POA: Preoptic area
PPa: Peduncular part of paraventricular area
PRM: Periretromamillary region
PSPa: Peduncular part of subparaventricular area
PSTh: Para-subthalamic nucleus
p1: Prosomere 1
p2: Prosomere 2
p3: Prosomere 3
PT: Pretectum
PTh: Prethalamus
rf: Retroflex tract
RM: Retromamillary region
RP: Roof plate
RPa: Rostral paraventricular nucleus
RTu: Retrotuberal area
SCH: Suprachiasmatic nucleus
SPa: Subparaventricular area
SPall: Subpallium
STh: Subthalamic nucleus
T/ATD: Acroterminal domain of the tuberal region
Tel: Telencephalon
Th: Thalamus
THy: Terminal hypothalamus
TPa: Terminal part of paraventricular area
TSPa: Terminal part of subparaventricular area
TSO: Terminal supraoptic nucleus
Tu: Tuberal region
TuD: Dorsal tuberal domain
TuI: Intermediate tuberal domain
TuSbO: Tuberal suboptic nucleus
VM: Ventromedial hypothalamic nucleus
VMc: Core portion of the VM
VMs: Shell portion of the VM
VPa: Ventral portion of the peduncular paraventricular area
VPM: Ventral premamillary nucleus.
